# Improved delineation of CT virtual bronchoscopy by ultrahigh-resolution CT: comparison among different reconstruction parameters

**DOI:** 10.1007/s11604-020-00972-y

**Published:** 2020-04-15

**Authors:** Takuya Adachi, Haruhiko Machida, Makiko Nishikawa, Takahiro Arai, Toshiya Kariyasu, Masamichi Koyanagi, Kenichi Yokoyama

**Affiliations:** 1 Department of Radiology, Kyorin University Hospital, 6-20-2 Shinkawa, Mitaka, Tokyo 181-8611 Japan; 2 Department of Radiology, Faculty of Medicine, Kyorin University, 6-20-2 Shinkawa, Mitaka, Tokyo 181-8611 Japan

**Keywords:** CT virtual bronchoscopy, Maximal recognizable bronchial bifurcation order, Peripheral pulmonary lesions, Ultrahigh-resolution CT

## Abstract

**Purpose:**

We compared the maximal recognizable bronchial bifurcation order (MRBBO) in CT virtual bronchoscopy (CTVB) using ultrahigh-resolution CT (UHRCT) and different reconstruction parameters.

**Materials and methods:**

We enrolled 38 patients undergoing noncontrast chest CT by UHRCT and reconstructed CTVB utilizing 3 different combinations of reconstruction parameters, as classified into Group A (matrix size, 512; slice thickness, 1.0 mm), Group B (matrix size, 512; slice thickness, 0.5 mm), and Group C (matrix size, 1024; slice thickness, 0.25 mm). In right S1, left S1 + 2, and both S3 and S10, two reviewers counted the number of consecutively identified bronchial bifurcations to compare MRBBO among these groups using Kruskal–Wallis test.

**Results:**

In these segments, MRBBO increased from Group A to C. MRBBO was significantly higher in Group C than in both Groups A and B in all the segments except left S10 (*P* < 0.05 for all). In left S10, it was significantly higher in Group C than in Group A (*P* < 0.05) but comparable between Groups B and C (*P* = 0.122).

**Conclusions:**

MRBBO is higher in CTVB by UHRCT utilizing 1024-matrix size and 0.25-mm thickness than parameters currently recommended for CTVB (matrix size, 512; slice thickness, 0.5–1.0 mm).

## Introduction

Widespread use of multidetector computed tomography (MDCT) scanners has increased the incidental detection of peripheral pulmonary lesions (PPLs) subsequently diagnosed by surgical, percutaneous needle, or transbronchial biopsy. The transbronchial method offers the lowest complication rate but can require tough insertion of a bronchoscope and/or a biopsy instrument into the lesions [1].

In transbronchial biopsy, CT virtual bronchoscopy (CTVB) is commonly used for navigation to assist scope insertion and thus to improve diagnosis of PPLs because of its 3-dimensional delineation of the tracheal and bronchial lumina as observed by actual bronchoscopy [2–4]. The recent introduction of an ultrathin bronchoscope (external diameter, ≤ 3 mm) in CTVB navigation has required higher spatial resolution to improve delineation of small peripheral bronchi [5, 6]. To address this need and improve in- and through-plane spatial resolution of CT images in clinical settings, ultrahigh-resolution CT (UHRCT) scanners have been introduced [7, 8]. However, we believe their utility for CTVB has not been reported.

We therefore undertook this pilot study to compare the maximal recognizable bronchial bifurcation order in CTVB by UHRCT using different reconstruction parameters and assessed whether use of UHRCT improved delineation of CTVB compared to that obtained using standard MDCT.

## Materials and methods

### Study population

We retrospectively identified 88 consecutive adult patients who underwent noncontrast chest CT using a UHRCT scanner (Aquilion Precision; Canon Medical Systems, Tokyo, Japan) with superhigh-resolution (SHR) scan mode (slice thickness, 0.25 mm; number of detector channels, 1792) from April 1 through May 31, 2017 at our institution. We excluded 50 of the 88 whose image data were deemed invalid for analysis because of significant CT image artifacts due to poor breath-hold (*n* = one), inadequate positioning of the upper limb (one), and pulmonary or bronchial structural distortions due to post-operative (*n* = 13), interstitial pneumonia (13), pulmonary emphysema (six), bronchiectasis (five), post-radiotherapy state (four), non-tuberculous mycobacterial infection (two), elevated diaphragm (two), pneumonia (one), bronchial obstruction (one), and traumatic hemopneumothorax (one). Thus, the final study population comprised 38 patients (17 men, 21 women; aged 24–89 years, mean age 63 ± 15 years) with mean body weight of 56.6 ± 9.9 kg (range 37.4–77.8 kg) and body mass index (BMI) of 22.7 ± 3.4 kg/m^2^ (range 16.9–30.7 kg/m^2^).

Our institutional review board approved this retrospective study, and we obtained written informed consent from all patients.

### CT scan technique

Patients underwent standard noncontrast routine helical chest CT scanning covering the entire lungs in a cranio-caudal direction during breath-hold using the UHRCT scanner with the SHR scan mode (slice collimation, 0.25 mm × 160 rows; number of channels, 1792). Scan parameters were: tube voltage, 120 kV; noise index, 12 Hounsfield units (HU) for the 5-mm reconstruction in filtered back projection by automatic exposure control; helical pitch, 0.806; rotation time, 0.5 s; and x-ray focus size, 0.4 × 0.5 mm or 0.6 × 0.6 mm. Just before each CT scanning, we checked the maximal tube current displayed on the CT console and selected the focus size of 0.4 × 0.5 mm unless the tube current exceeded 260 mA because the maximal limitation of tube current is 260 mA for the focus size of 0.4 × 0.5 mm and 310 mA for that of 0.6 × 0.6 mm.

We recorded the volume CT dose index (CTDI_vol_, measured in mGy) and dose length product (DLP, measured in mGy・cm) displayed on the dose report on the CT scanner for each patient and calculated the mean CTDI_vol_ and DLP for all patients.

### CTVB image generation

For each patient, we used adaptive iterative dose reduction (AIDR 3D Enhanced Strong; Canon Medical Systems) to reconstruct the UHRCT image datasets with field of view of 320–375 mm and a kernel for mediastinal display (FC03) utilizing 3 different combinations of reconstruction parameters including matrix size (512^2^ and 1024^2^) and slice thickness/interval (0.25/0.2, 0.5/0.4, and 1.0/0.8 mm) (Table 1).

**Table 1:** Three combinations of reconstruction parameters

Group	Matrix size	Slice thickness (mm)	Slice interval (mm)
A	512 × 512	1.0	0.8
B	512 × 512	0.5	0.4
C	1024 × 1024	0.25	0.2

**Fig. 1:**
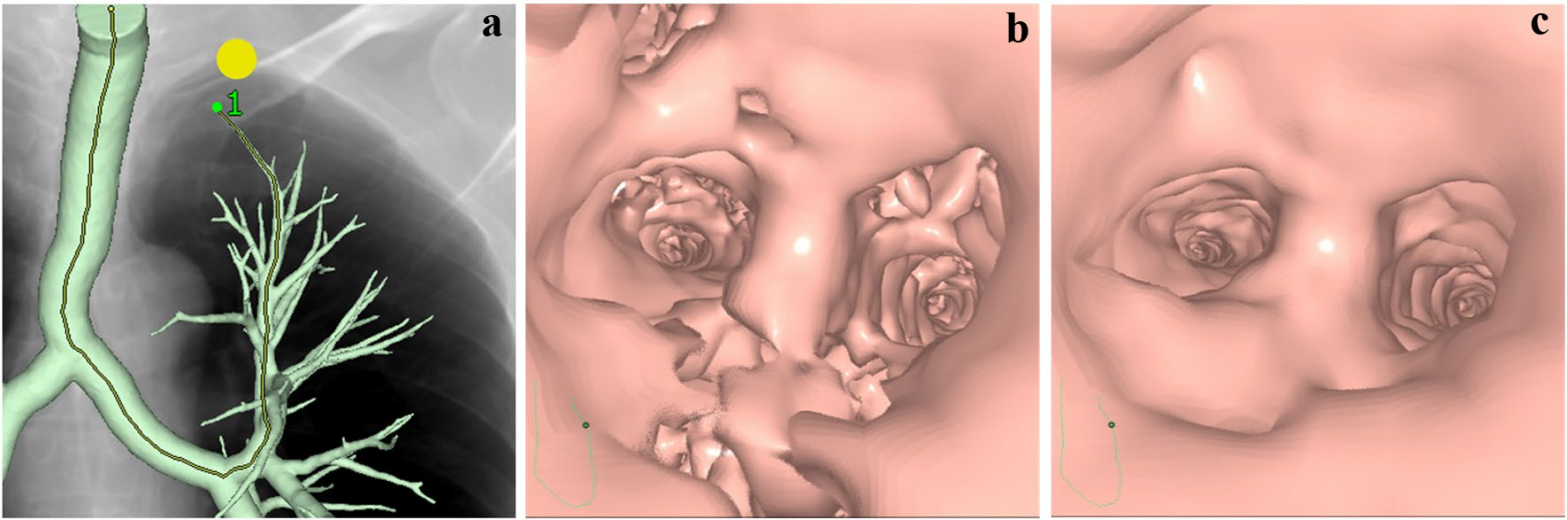
Process of image generation for computed tomographic (CT) virtual bronchoscopy (CTVB). A pulmonary nodule (yellow circle) is simulated as a target lesion adjacent to the pleura of the most apical part in Segment 1 + 2 of the left lung for CTVB on a simulated chest radiograph, and a tracking line (yellow line) is then automatically drawn running through the center of the tracheal and bronchial lumina to the nodule on the frontal overlapped view of a volume-rendered bronchial tree (**a**). The wall region of the tree has been automatically extracted and the simulated chest radiograph reconstructed from the same CT volume data. Endoscopic view of CTVB before adjustment of the threshold to preserve continuity of the inner surface of the bronchial tree through the entire route (**b**). Endoscopic view of CTVB after threshold adjustment shows improved continuity (**c**)

All datasets were transferred to a dedicated workstation (SYNAPSE VINCENT version 4.6.0007; FUJIFILM Medical, Tokyo, Japan), on which an experienced radiology technologist reconstructed images by: applying an automatic algorithm to extract the region of the bronchial wall to generate 3-dimensional CTVB; simulating a pulmonary nodule as a target lesion adjacent to the pleura of the most apical part in Segment 1a of the right lung (right S1) and Segment 1 + 2a of the left lung (left S1 + 2) and the most anterior part of Segment 3b of the right lung (right S3) and Segment 3b of the left lung (left S3) (both S3) and the most basal part in Segment 10c of the right lung (right S10) and Segment 10c of the left lung (left S10) (both S10) in each patient; applying an automatic function to draw a tracking line running through the center of the tracheal and bronchial lumina to the nodule under each combination of reconstruction parameters; and adjusting the threshold to preserve continuity of the inner surface through the entire route under each condition (Fig. 1). We classified the CTVB image sets with the 3 different combinations of reconstruction parameters into Groups A to C, with C having the smallest voxel size (Table 1).

### CTVB assessment

Using a CTVB navigation mode, the experienced radiology technologist and a board-certified radiologist observed the CTVB images along the tracking line toward the simulated nodules in the right S1, left S1 + 2, and both S3 and S10 using a paging method to confirm the absence of pathology throughout the route for each patient. The 2 blinded reviewers then assessed all the CTVB image sets of Groups A to C in random order, and in consensus, they counted the number of consecutively identified bronchial bifurcations (based on the carina as the first bifurcation) to determine the maximal recognizable bronchial bifurcation order in the right S1, left S1 + 2, and both S3 and S10 for each group. The reviewers confirmed identification of bronchial bifurcation when they could clearly observe at least 2 bronchial orifices as they moved back and forth at least 3 times.

### Statistical analysis

All continuous variables were expressed as mean ± standard deviation (SD). We analyzed statistics using commercially available software (IBM SPSS Statistics, version 23 IBM SPSS, Armonk, NY, USA). We used Kruskal–Wallis test to compare the maximal recognizable bronchial bifurcation order in the right S1, left S1 + 2, and both S3 and S10 among Groups A, B, and C. We selected Groups A and B as counterparts to Group C because Group C had the smallest voxel size, and the current recommendation for slice thickness for CTVB using standard MDCT scanners with 512^2^ matrix size is 0.5–1.0 mm [2]. In each segment and group, we used Spearman’s rank correlation coefficient to assess correlation between the maximal recognizable bronchial bifurcation order and BMI. A *P* value less than 0.05 was considered statistically significant.

## Results

We observed a mean CTDI_vol_ of 12.8 ± 1.5 mGy and mean DLP of 581.6 ± 93.1 mGy cm.

**Fig. 2:**
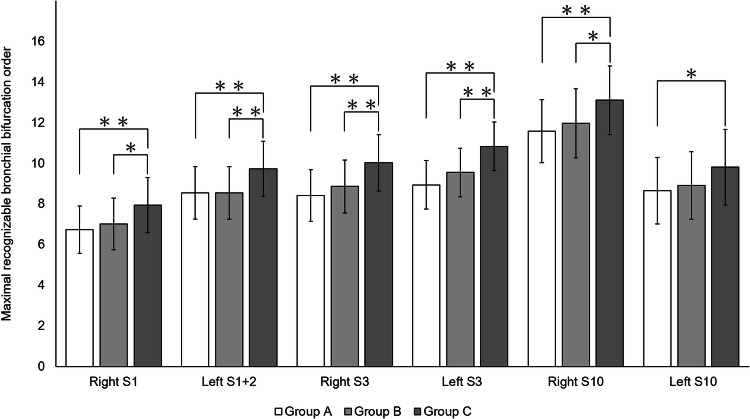
Bar graphs show the maximal recognizable bronchial bifurcation order on computed tomographic (CT) virtual bronchoscopy (CTVB) obtained using various reconstruction parameters in Segment 1a of the right lung (right S1), Segment 1 + 2a of the left lung (left S1 + 2), Segment 3b of the right lung (right S3), Segment 3b of the left lung (left S3), Segment 10c of the right lung (right S10), and Segment 10c of the left lung (left S10). In all these segments, the maximal recognizable bronchial bifurcation order increased from Group A to Group C. Asterisk indicates statistically significant differences by Kruskal–Wallis test between each combination (^∗^*P* < 0.05 and ^∗∗^*P* < 0.01, respectively)

**Fig. 3:**
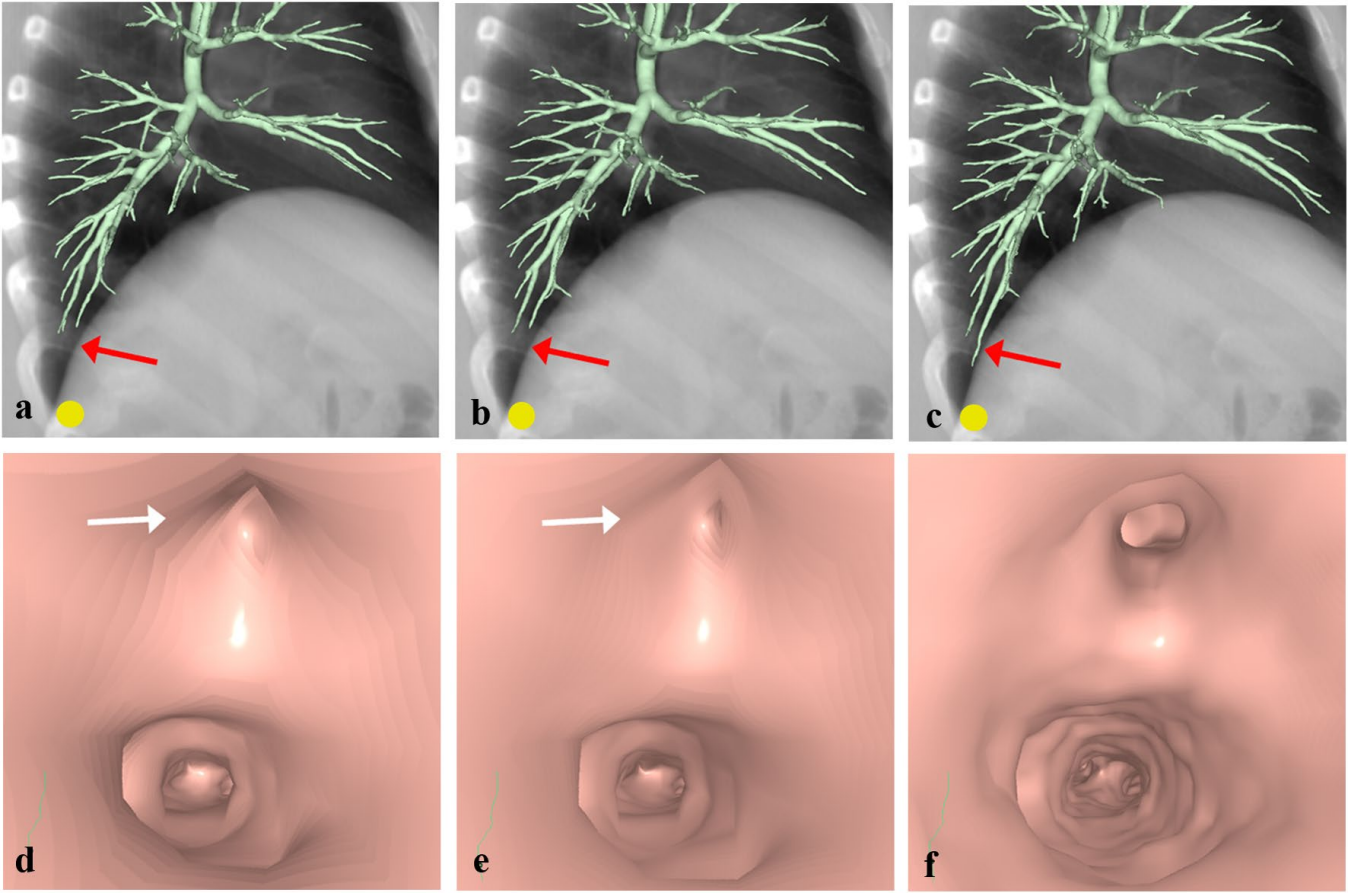
Computed tomographic (CT) virtual bronchoscopy (CTVB) for a simulated nodule (yellow circle) adjacent to the pleura of the most basal part in Segment 10c of the right lung in a 73-year-old man. On the lateral overlapped view of a volume-rendered bronchial tree and a simulated chest radiograph reconstructed from the same CT volume data (**a**–**c**), delineation of peripheral bronchi in this segment (red arrows) improved from Group A (**a**) to Group B (**b**) to Group C (**c**). On the endoscopic view of CTVB at the 13th bifurcation in the segment (**d**–**f**), 2 bronchial orifices are clearly identified in Group C (**f**), but one of these orifices appears to be obstructed (white arrows) in Groups A (**d**) and B (**e**). Detailed delineation of the bronchial inner surface is also better in Group C (**f**) than in Groups A (**d**) and B (**e**)

The maximal recognizable bronchial bifurcation order tended to increase from Group A to Group C in the right S1, left S1 + 2, and both S3 and S10 (Table 2 and Fig. 2). The maximal recognizable bronchial bifurcation order was significantly higher in Group C than in both Groups A and B in all these segments except left S10 (*P* < 0.05 for all); was significantly higher in Group C than in Group A (*P* = 0.021) but comparable between Groups B and C in left S10 (*P* = 0.122) (Fig. 2). All of these values in Group C were higher by one or more than in Groups A and B. Figure 3 shows the improved delineation of peripheral bronchi and bronchial orifices at the maximal recognizable bronchial bifurcation order in each patient in Group C compared with delineation in Groups A and B in CTVB images obtained using UHRCT. No significant correlation was found between the maximal recognizable bronchial bifurcation order and BMI in any segments and groups (*P* = 0.320–0.989, *ρ* =  − 0.191–0.188).

**Table 2:** Comparison of maximal recognizable bronchial bifurcation order among Groups A, B and C

	Group A	Group B	Group C
Right S1	6.7 ± 1.2	7.0 ± 1.3	7.9 ± 1.4
Left S1 + 2	8.6 ± 1.3	8.6 ± 1.3	9.7 ± 1.4
Right S3	8.4 ± 1.3	8.9 ± 1.3	10.0 ± 1.4
Left S3	8.9 ± 1.2	9.6 ± 1.2	10.8 ± 1.2
Right S10	11.6 ± 1.5	12.0 ± 1.7	13.1 ± 1.7
Left S10	8.7 ± 1.6	8.9 ± 1.7	9.8 ± 1.9

*Left S1 + 2* Segment 1 + 2a in the left lung, *Left S3* Segment 3b in the left lung, *Left S10* Segment 10c in the left lung, *Right S1* Segment 1a in the right lung, *Right S3* Segment 3b in the right lung, *Right S10* Segment 10c in the right lung

## Discussion

As expected, we observed the highest maximal recognizable bronchial bifurcation order in CTVB by UHRCT utilizing matrix size of 1024^2^ and slice thickness of 0.25 mm, and that order was significantly higher than that obtained using the values currently recommended for CTVB using standard MDCT scanners (matrix size, 512^2^; slice thickness, 0.5 or 1.0 mm) [2]. The UHRCT scanner used in our study has been in clinical application since 2017 and achieved higher spatial resolution (maximal spatial resolution, approximately 0.15 mm or less) than that of standard MDCT scanners, even with the same voxel size [7–10]. Physical specifications improved by UHRCT included the SHR scan mode (slice thickness, 0.25 mm; number of channels, 1792) and smaller x-ray tube focus (smallest, 0.4 × 0.5 mm). In fact, delineation of the anatomy of the temporal bone has been reported more conspicuous utilizing the improved detector of UHRCT than depiction achieved using standard MDCT, even with the same voxel size [11]. In addition, UHRCT facilitates the use of smaller voxel size to decrease partial volume averaging, so the superiority of CTVB by UHRCT to that utilizing standard MDCT has been shown in delineating more distal bronchi while preserving the continuity of the bronchial inner surface [4, 6].

The maximal recognizable bronchial bifurcation order by UHRCT ranged from 7.9 ± 1.4 to 13.1 ± 1.7 (median, 10; mean, 10.2 ± 2.4) in Group C, higher than that reported by standard MDCT [4, 6]. Specifically, in the study by Asano and colleagues, the median order was 6 using 16- or 64-detector-row CT with matrix size of 512^2^ and slice thickness of 0.5 to 1.0 mm; in the study by Khan and colleagues, the mean order was 6.5 ± 0.3 using 16-detector-row CT with matrix size of 512^2^ and slice thickness of 0.75 mm. An ultrathin bronchoscope allows more distal insertion than a larger conventional bronchoscope with external diameter of approximately 5 to 6 mm, and maximal insertion of the thinner scope to the ninth order has been reported (median, fifth order) [4]. Thus, use of UHRCT can better assist this maximal insertion of the ultrathin bronchoscope. For transbronchial biopsy, diagnostic yield can be improved and examination time and risk of complication reduced by insertion of an ultrathin bronchoscope to PPLs with the aid of CTVB navigation by UHRCT employing matrix size of 1024^2^ and slice thickness of 0.25 mm [1, 4].

The maximal recognizable bronchial bifurcation order was higher in the left S1 + 2 than in the right S1, presumably because the bronchial anatomy tends to detour to the apex more prominently in the left S1 + 2. Image noise and beam-hardening artifact caused by surrounding bony structures in this apical area might diminish bronchial delineation compared with both S10. However, the use of UHRCT in combination with model-based iterative reconstruction can improve the maximal recognizable bronchial bifurcation order in this apical region. We excluded from analysis a patient with poor breath-hold but did not perform electrocardiographically gated chest CT scanning, which offers higher radiation exposure to patients. Thus, the maximal recognizable bronchial bifurcation order was lower in the left S10 than the right S10 and comparable between Groups B and C only in the left S10, presumably because bronchial delineation might be more susceptible to motion artifacts from cardiac pulsation in the left S10. According to the vendor of our workstation, its automated tracking function permits the automatic drawing of a tracking line into bronchi with inner diameter of at least one mm. Thus, improvement of this function will even further increase the maximal recognizable bronchial bifurcation order in CTVB by UHRCT.

### Study limitations

Our study was limited because it was retrospective and included only a small study population at a single institution, and we restricted our pilot assessment of maximal recognizable bronchial bifurcation order to only the right S1, left S1 + 2, and right and left S3 and S10, whereas we selected the right S1 and left S1 + 2 as the most apical segments, both S10 as the most basal segments, and both S3 where the bronchi run almost parallel to the axial CT plane. We did not use actual bronchoscopy as a reference to confirm delineation of bronchial orifices, and insertion of even an ultrathin bronchoscope to the maximal recognizable bronchial bifurcation order delineated using CTVB navigation by UHRCT may not be possible [4]. Confirmation of the clinical utility of CTVB navigation by UHRCT to assist actual bronchoscopy and thus transbronchial biopsy may warrant a large-scale multicenter prospective study. Further, we used the only workstation at our institution that was capable of generating CTVB by UHRCT with matrix size of 1024^2^ or more, but its limited capacity to process high-volume data did not permit reconstruction of UHRCT images with maximal matrix size of 2048^2^. Our findings may also have been influenced by the smaller body weight and BMI of our Japanese patients compared to that of average-sized patients in Western countries, and the noise index in our study was that commonly used for routine chest CT at our institution and might be relatively small. Nevertheless, both the CTDI_vol_ and DLP complied with the criteria for radiation dose to patients for standard chest CT (CTDI_vol_, 30 mGy; DLP, 650 mGy cm) according to European guidelines on quality criteria for CT [12]. The lower radiation dose may have affected our results by increasing image noise, whereas more advanced reconstruction techniques for further reducing image noise, such as model-based iterative reconstruction, are applicable. The 2 blinded reviewers in consensus assessed the CTVB image sets, which may result in a confirmation bias.

## Conclusion

In conclusion, with preserving continuity of the bronchial inner surface, CTVB by UHRCT using matrix size of 1024^2^ and slice thickness of 0.25 mm improves delineation of bronchial bifurcation compared to that achieved using the values currently recommended for CTVB (matrix size, 512^2^; slice thickness, 0.5–1.0 mm), and its clinical application for navigation in transbronchial biopsy, particularly using an ultrathin bronchoscope, may improve clinical management for patients with PPLs.
